# Digital twin for personalized medicine development

**DOI:** 10.3389/fdgth.2025.1583466

**Published:** 2025-08-07

**Authors:** Saniya Y. Saratkar, Meher Langote, Praveen Kumar, Pradnyawant Gote, Induni Nayodhara Weerarathna, Gaurav V. Mishra

**Affiliations:** ^1^ Department of Artificial Intelligence and Data Science, Faculty of Engineering and Technology, Datta Meghe Institute of Higher Education and Research, Wardha, India; ^2^ Department of Computer Science Medical Engineering, Faculty of Engineering and Technology, Datta Meghe Institute of Higher Education and Research, Wardha, India; ^3^ Department of Computer Science and Departments, Faculty of Engineering and Technology, Datta Meghe Institute of Higher Education and Research, Wardha, India; ^4^ Department of Biomedical Sciences, Allied Health Sciences, Datta Meghe Institute of Higher Education and Research, Wardha, India; ^5^ Department of Radiodiagnosis, Datta Meghe Institute of Higher Education and Research, Wardha, India

**Keywords:** digital twin, personalized medicine, artificial intelligence, healthcare, internet of things (IoT)

## Abstract

Digital Twin (DT) technology is revolutionizing healthcare by enabling real-time monitoring, predictive analytics, and highly personalized medical care. As a key innovation of Industry 4.0, DTs integrate advanced tools like artificial intelligence (AI), the Internet of Things (IoT), and machine learning (ML) to create dynamic, data-driven replicas of patients. These digital replicas allow simulations of disease progression, optimize diagnostics, and personalize treatment plans based on individual genetic and lifestyle profiles. This review explores the evolution, architecture, and enabling technologies of DTs, focusing on their transformative applications in personalized medicine (PM). While the integration of DTs offers immense potential to improve outcomes and efficiency in healthcare, challenges such as data privacy, system interoperability, and ethical concerns must be addressed. The paper concludes by highlighting future directions, where AI, cloud computing, and blockchain are expected to play a pivotal role in overcoming these limitations and advancing precision medicine.

## 1 Introduction

### 1.1 Introduction to DT technology in healthcare

DT technology is increasingly transforming the healthcare sector by offering advanced capabilities in predictive diagnostics, personalized treatment, workflow optimization, and medical education (1). A DT refers to a virtual replica of a real-world entity or system that is continuously updated with real-time data. This real-time synchronization allows for dynamic simulations and informed decision-making (2). In healthcare, DTs create virtual models of individual patients by combining their health data, medical history, lifestyle, and genetic information, helping doctors to foresee how diseases will progress, improve treatment methods, and tailor care to each patient (3). The integration of DTs with AI, the IoT, and ML provides a robust framework for enhancing healthcare delivery and outcomes (4).

### 1.2 Significance of PM and the role of DTs

PM aims to tailor medical care to the unique characteristics of each patient by leveraging genetic, environmental, and lifestyle factors (5). Traditional treatment models often follow a generalized “one-size-fits-all” approach, which fails to account for interindividual variability (6). In contrast, the use of DTs enables a shift toward precision care by creating individualized virtual models that can simulate responses to specific therapies, adjust interventions in real time, and foresee complications before they manifest (7). This synergy between DTs and PM supports the broader vision of predictive, preventive, and participatory healthcare, allowing for interventions that are not only more effective but also more resource-efficient (8).

### 1.3 Market trends and global adoption

The adoption of DT technology in healthcare is gaining strong momentum globally (9). Recent industry reports suggest that 66% of healthcare executives plan to invest in DT technologies over the next three years. The global market is projected to grow at a compound annual growth rate exceeding 30% between 2023 and 2027 (10). In India alone, the market is forecasted to expand from USD 800 million in 2023 to over USD 12 billion by 2032, reflecting growing interest in emerging economies (11). Additionally, around 25% of all digital transformation initiatives in healthcare are expected to incorporate DTs by 2025 (12). These numbers show that people involved in healthcare believe in the benefits of DTs, mainly because they can enhance patient care, make better use of resources, and lower healthcare expenses by using real-time data (13).

### 1.4 Evolution and historical background of DT technology

**Figure 1:**
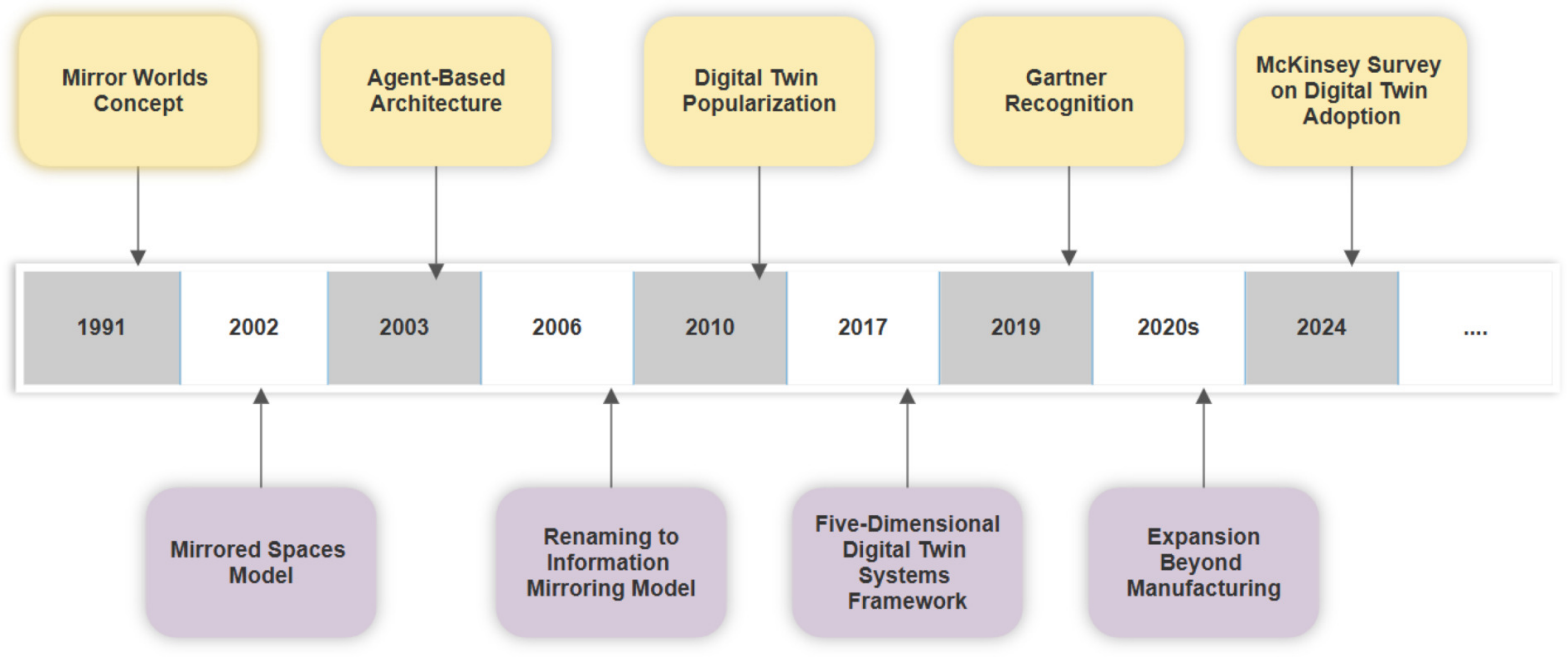
Evolutionary timeline of DT technology.

Understanding the historical context of DTs is essential to appreciate their current application in healthcare (14). The foundational concept dates back to the National Aeronautics and Space Administration (NASA)'s Apollo program in the 1970s, where ground-based simulators were used to replicate spacecraft behavior for mission planning and troubleshooting (15). The term “digital twin” was officially coined by NASA in 2002 in the context of product lifecycle management. Over time, DTs evolved from static digital models to dynamic, AI-integrated systems capable of mirroring complex and adaptive systems (16). While early applications were concentrated in aerospace and industrial manufacturing, the healthcare sector began adopting DT concepts around 2016 (17). The COVID-19 pandemic further catalyzed their use, especially in telemedicine, hospital capacity planning, and infectious disease modeling (18). As of 2024, it is reported that 86% of organizations across various industries view DTs as a core component of their digital innovation strategies (19) (20, 21),. This historical evolution underscores the maturity and adaptability of DT technology as it transitions into mainstream medical applications (22, 23). Figure 1 depicts the evolutionary timeline of DT technology.

This review will examine the current landscape of DT technology in healthcare, with a specific focus on its applications in predictive and PM. It highlights foundational principles and recent innovations, practical implementations, and addresses the challenges and ethical concerns. The review also discusses the potential of DTs to revolutionize clinical decision-making and patient care in a data-driven medical ecosystem. Further, the manuscript distinguishes between “personalized digital twins,” individualized virtual models continuously updated with patient-specific data, and “precision digital twins,” which simulate optimized treatments for stratified patient groups based on shared genetic or phenotypic features. Similarly, authors differentiate between “personalized medicine,” focused on tailoring clinical interventions, and “personalized health,” a broader concept encompassing lifestyle, prevention, and wellness strategies aligned to the individual (22, 24, 25). Table 1 shows the definitions of key terms used throughout this review, including distinctions between personalized and precision DTs, as well as between PM and personalized health.

**Table 1:** The definitions of key terms used throughout this review.

Sr. no	Term	Definition
1	Personalized DT	A virtual replica of an individual patient that integrates real-time, patient-specific data (e.g., genomics, vitals, lifestyle) to simulate and personalize diagnosis, treatment, and disease progression.
2	Precision DT	A DT model designed for a specific subgroup of patients (e.g., based on shared genetic markers or conditions) to simulate optimized, evidence-based interventions.
3	PM	A clinical approach that tailors medical decisions, treatments, and diagnostics to an individual based on genetic, biomarker, environmental, and lifestyle factors.
4	Personalized Health	A broader concept that includes personalized medicine but also focuses on wellness, disease prevention, health monitoring, and behavior modification tailored to individual needs.

## 2 Principles of digital twinning

### 2.1 Core concepts

The DT is a digital or virtual model that is created and updated in real-time to accurately represent the state of its physical twin at any given moment. Same as storing history data. These are not only DTs that mimic his physical twins, but the changes in DT are imitated by physical twins (20). DT technology is radically changing how patient care is delivered and quickly becoming disruptive in the medical field. Through real-time data integration, advanced analytics, and virtual simulation, DT paves the way for improved patient care, predictive analytics, clinical workflow optimization, training, and simulations (26, 27).

Real-time data integration involves the ongoing and instantaneous gathering and processing of information from multiple sources. DT technology pertains to the smooth transfer of data from tangible entities (such as medical devices, patient monitors, or sensors) to their digital equivalents. This type of integration facilitates immediate monitoring, analysis, and judgment, resulting in more precise forecasts and swift responses to alterations in the physical environment. Preserving correspondence between an actual thing and its DT is called “synchronization” between physical and digital entities (28).

#### 2.1.1 Principles and components of DT

The basic principles help combine digital and physical systems using DT, making complex tasks, like real-time synchronization and two-way data flow, much simpler. For instance, real-time synchronization functions by providing accurate instant mirroring from the physical replica to the virtual replica. Data is also moving bi-directionally for actionable insight and automated decisions (20, 29). For instance, in the health sector, DT adds real-time data from patient monitors to dynamically monitor healthcare metrics and alter treatments for further improvement. Another important property is dynamic learning, which enables the DT to gain knowledge and progress through processing incoming data. This primarily applies to applications such as PM, which involves the DT analyzing patient data to propose customized therapies. A DT contains mainly three parts: the physical entity, the digital replica, and the data and communication network (25).

A physical entity is a system or thing found in the real world, such as a structure, manufacturing machine, or human organ. The digital copy is the virtual equivalent that imitates the real thing, displaying its structure, actions, and output. Lastly, the data and communication network connect the entities, enabling real-time data collection, transfer, and analysis. When taken as a whole, these elements allow digital twins to provide critical insights, improve performance, and stimulate innovation across various sectors (30).

### 2.2 Key properties

**Figure 2:**
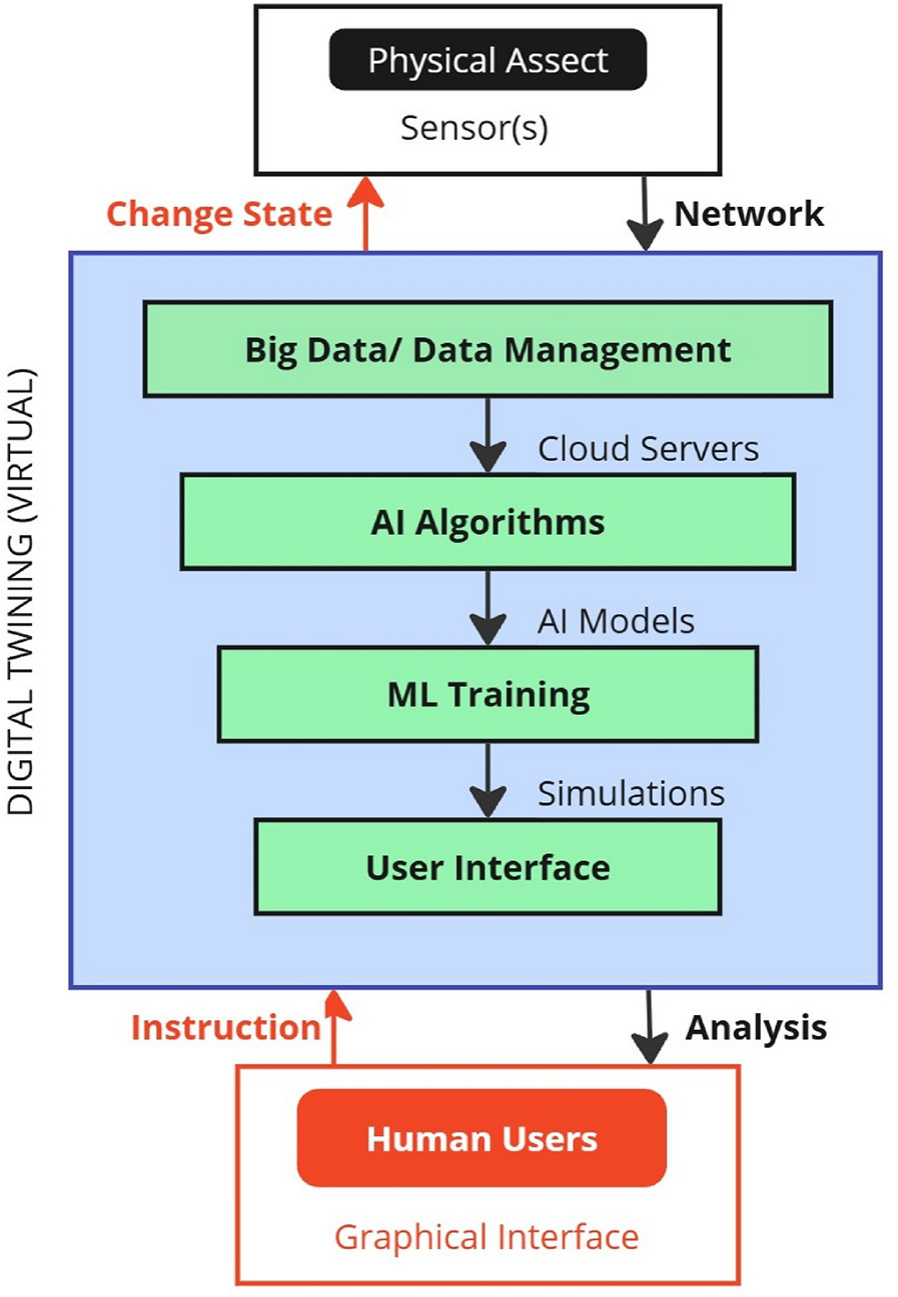
Information flow among physical assets, DTs, and users. Adapted with permission from “The flow of information between physical asset, digital twin and human users” by Suresh Neethirajan and Bas Kemp licensed under CC BY 4.0.

DTs are virtual replicas of physical assets (31). Real-time updates regarding the physical or biological entity's interactions with its surroundings, workload, and numerous other factors are necessary to simulate a physical asset and its behavior accurately. This necessitates a collection of sensors capable of sending and receiving data over a secure, isolated network or the internet. Additionally, it must collect, store, and process vast amounts of data in real-time, which requires significant computing, storage, and processing capabilities. It must leverage the most advanced innovations in big data, data management, and cloud computing (32). Thirdly, twins must be able to interpret sizable volumes of streaming data. Such an ability implies that the computational capacity of an individual is often surpassed, necessitating the use of AI algorithms to differentiate between valuable and incorrect information. Additionally, it involves using AI algorithms to provide suggestions and recommend actions. Fourthly, the twin has to be capable of investigating various cause-and-effect scenarios over time and applying insights to enhance the performance of physical assets. This task includes conducting numerous alternative scenarios, test cases, and simulations related to the matter. Once more, this refinement goes beyond human comprehension, indicating the need to train machine learning algorithms in specific contexts for optimal action plan training, experimentation, and the development of the best action strategies (33). Lastly, an interactive digital user interface must make this knowledge easily accessible to critical human decision-makers. Finally, a user-friendly digital interface should ensure that all this information is readily available to key human decision-makers. Figure 2 illustrates the information flow among physical assets, DTs, and users.

### 2.3 Key enabling technologies in DT

**Figure 3:**
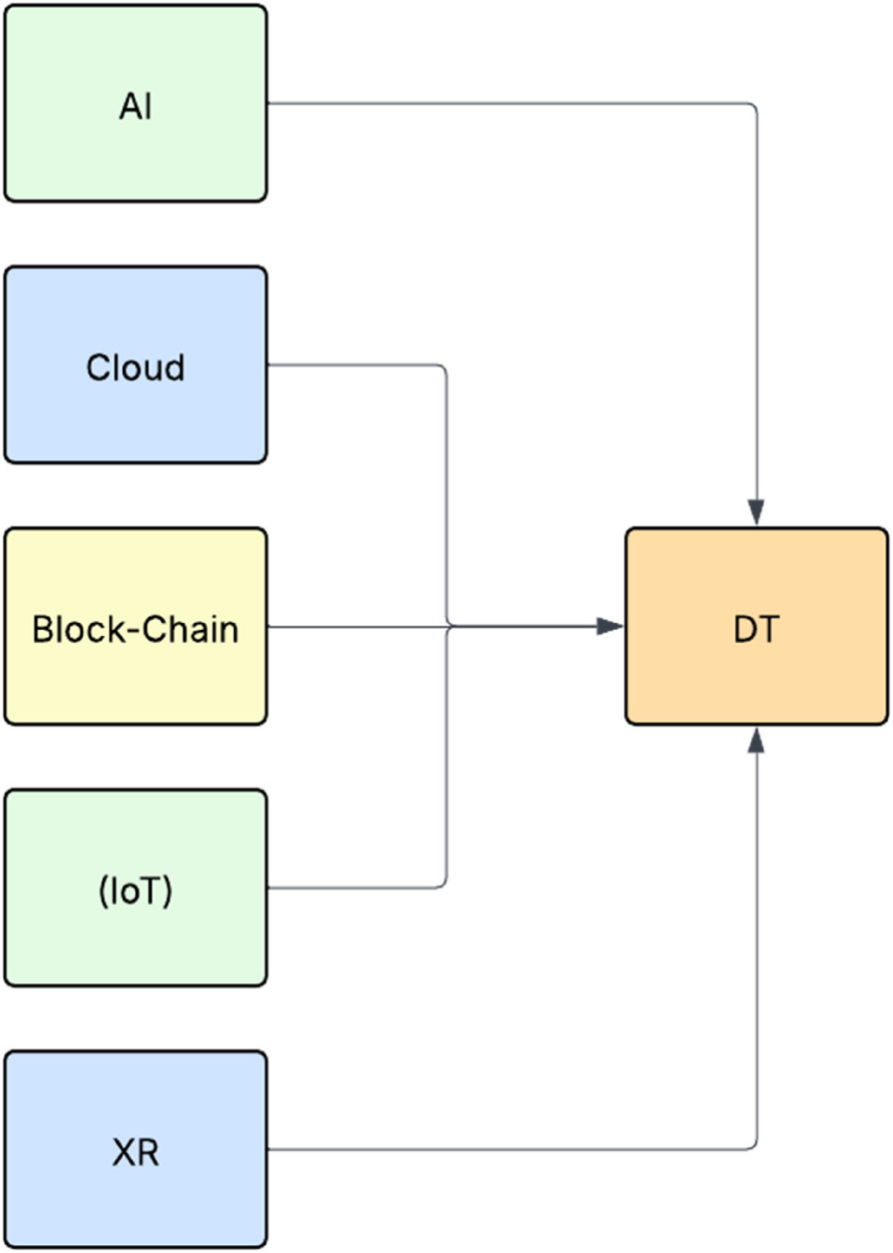
Technology integration with DT for improved efficiency.

DT leverages five primary technologies to collect and preserve real-time data, acquire insights for valuable decision-making, and generate a digital replica of a tangible object (Figure 3). Cloud computing offers hosted services, AR and VR bring digital twins to life, AI accelerates processes in real-time, and the IoT has emerged as a crucial technology. Furthermore, the extent to which DT leverages specific technology, such as blockchain, varies based on the application (34). Each of these technologies significantly enhances the capabilities of DTs across numerous sectors.

#### 2.3.1 IoT

IoT serves as the key technology for enhancing digital transformation. It pertains to an extensive system of interrelated “things.” Connections can occur among various devices, between individuals and devices, or among individuals themselves. IoT is essential to Industry 4.0, driving digital transformation by connecting people, products, and processes through networked smart sensors and actuators. Businesses may connect and manage devices, analyze industrial data, boost productivity, boost income, and optimize operations in both manufacturing and field settings by leveraging IoT platforms. As a result, expenses are decreased, and more savings are generated (35). By 2027, it's predicted that over 90% of IoT systems will have DT capabilities. IoT continuously collects, processes, updates, and synchronizes data from physical things via sensors. The information the IoTs provide can generate a digital representation of a physical object. IoT and DTs work together to improve operational efficiency, reduce downtime, and facilitate proactive decision-making based on data patterns (36).

#### 2.3.2 Cloud computing

Cloud computing is crucial for DT technology, as it supplies the infrastructure required for processing, storing, and analyzing massive amounts of data. Manufacturers can efficiently store and retrieve data online thanks to its hosted services. Processes are accelerated by cloud technology, which also helps businesses become more agile, secure, collaborative, and cost-effective. It gives DT cloud-based data preparation and storage options, enabling them to handle large amounts of data, reduce calculation time, and access essential data from any location (37). Cloud solutions can produce sophisticated, captivating visual representations that simplify manufacturing lines or operations. Furthermore, cloud technologies improve DTs by enabling concurrent model interaction in virtual and real-world environments. Cloud services empower DTs to evolve beyond digital copies of physical entities to deliver new functionalities that remove technological limitations, promoting real-time collaboration within manufacturing environments. Businesses like Microsoft provide cloud features through comprehensive end-to-end solutions, including Azure Cortana Intelligence, Microsoft HoloLens, Microsoft Azure IoT, and the Microsoft Azure Big Compute portfolio. These cloud-based solutions give manufacturers the resources to adopt DTs and profit from their many advantages quickly. Cloud computing's distributed architecture improves DT management by providing enterprises with instant access to scalable and flexible resources. Without the need for substantial on-site resources, various cloud services, like Platform as a Service (PaaS) and Infrastructure as a Service (IaaS), simplify the deployment and integration of DT into current systems (38).

#### 2.3.3 AI

The computer science discipline of AI aims to mimic the characteristics of intelligence to build robots that can react in ways comparable to human cognition. Some of its focus areas are neural networks, ML, deep learning (DL), robotics, image and speech recognition, and expert systems. AI enhances DT by offering advanced analytical capabilities to automatically assess incoming data, produce insightful conclusions, predict results, and recommend approaches to potential problems. It is emphasized that DT and AI technologies are essential elements of Industry 4.0. Through AI, DTs can effectively simulate complex real-world systems. Data obtained from IoT devices can be utilized to learn from and engage with actual manufacturing environments, revealing areas for enhancement and aiding tactical decision-making (37, 39).

#### 2.3.4 Extended reality (XR)

Immersion technologies like VR, AR, and mixed reality (MR) are all included in the broad term “XR” (40). These technologies improve our understanding of reality by combining the digital and physical (41). They provide significance and interaction with our surroundings by superimposing digital stuff on top of them. This cutting-edge technology supplies new methods for seeing and interacting with DT. XR technologies' immersive and interactive experiences can significantly enhance our interaction with digital data. Furthermore, XR technologies have the potential to significantly improve human relationships, particularly in contexts involving remote help and training. VR-enabled digital twins, for instance, enable engineers to model and test aircraft systems before actual deployment in the aerospace sector, cutting down on development time and cost (42).

#### 2.3.5 Blockchain technology (BT)

BT is a decentralized, continuously growing chain of records, or “blocks,” managed by several computers in a peer-to-peer network. Before each transaction is added to the BT, it is verified by several computers connected to the internet (43). Once this data has been processed, the BT makes sure that every computer in the network secures it at the same time, creating digital records that are unchangeable and permanent. This technology improves DT solutions by strengthening data security, privacy, and dependability (44).

### 2.4 Architecture of DT

**Figure 4:**
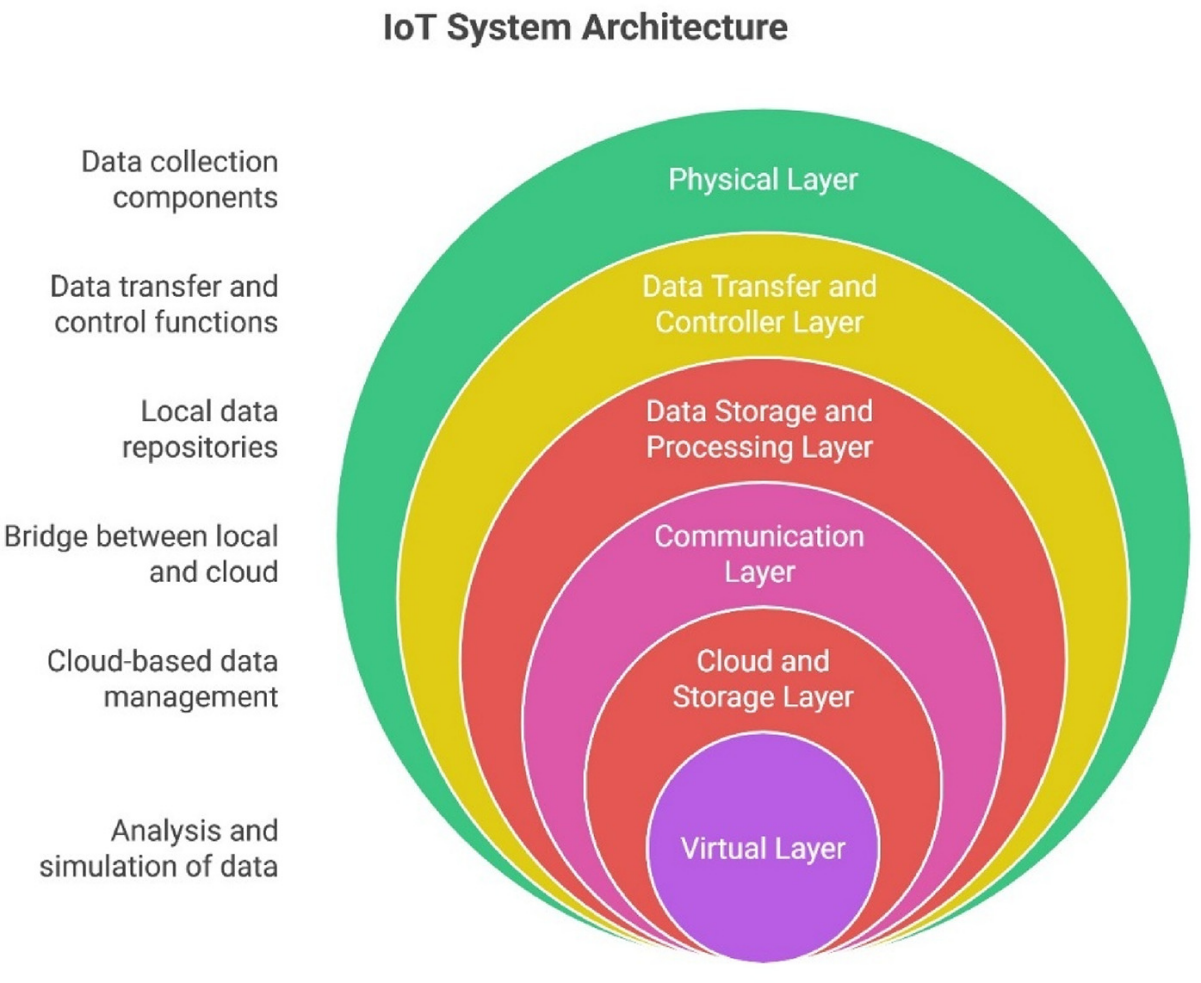
Structured six-layer architecture of DT.

The author of outlines a comprehensive six-layer architecture, while other authors have contributed to the five layers, leading to the presentation of the six-layer architecture (26). As illustrated in Figure 4, Reference (45) recommends a six-layer DT architecture to accomplish successful device integration with their virtual counterparts in the cyber-physical domain and to enable effective information interchange between DTs, physical entities, and the broader environment. The 5C paradigm is expanded upon by this six-layer framework (43). Developed before the phrase “DT,” the 5C paradigm incorporates cyber-physical systems (CPS) replicating physical systems in cyberspace. Five different phases make up the 5C model.

Intelligent connection, data transformation into information, cyber integration, cognitive capabilities, and architecture configuration. Sustainability (46).

The architecture of DT technology provides a robust framework that bridges the physical and virtual domains, enabling seamless interaction, monitoring, and decision-making (26). It integrates diverse components such as physical devices, sensors, communication protocols, and advanced simulation tools, making it a cornerstone of Industry 4.0 (47). There are two primary layers in DT technology (29): and (48).

#### 2.4.1 Layer 1: physical devices

This foundational layer comprises actuators, sensors, and other hardware elements embedded within the physical system. These devices continuously gather real-time data, such as temperature, pressure, speed, or vibration, to produce an accurate digital representation. For example, in a manufacturing plant, sensors installed on conveyor belts capture operational data for performance monitoring and fault detection. This layer serves as the sensory system of the DT, directly interfacing with the real-world entity (49).

#### 2.4.2 Layer 2: data source layer

Layer 2 specifies the data sources connected to the physical entity, facilitating structured data acquisition. It organizes raw data streams produced by Layer 1 to prepare them for processing in the following layers. This layer also facilitates device management, ensuring the synchronization and functionality of sensors and actuators. Examples include IoT gateways that aggregate data from different sensors.

#### 2.4.3 Layer 3: interface and communication layer

This layer acts as the gap between the physical and virtual domains. This involves local data vaults, where immediate data is stored for the sensors and controllers in Layer 2. OPC-UA (Open Platform Communication-Unified Architecture) is essential, ensuring safe and standardized communication among the devices and layers. For instance, in industrial automation, OPC-UA makes it easy to integrate a wide range of machines from various manufacturers into one system. This layer is essential because it ensures that data flows bidirectionally, where the DT will send control commands back to the physical system (50).

#### 2.4.4 Layer 4: data processing layer

Layer 4 is the data-to-information converter; raw data gets refined and processed to become actionable insight. IoT technologies improve this layer by supporting real-time analytics and edge computing. Converting raw sensor data into meaningful information connects the lower physical and upper decision-making layers. For instance, we can use vibration data from any machinery to anticipate wear and tear, allowing us to take appropriate maintenance actions before problems arise.

#### 2.4.5 Layer 5: cloud and data repository layer

This layer manages storing and organizing historical and real-time data in cloud-based repositories. Advanced data management systems enhance data availability, precision, and accessibility. This layer allows for complex analytics like trend analysis and predictive modeling to support long-term decision-making. This layer commonly uses tools like AWS IoT Analytics or Microsoft Azure DTs to manage and analyze large datasets.

#### 2.4.6 Layer 6: cognition and simulation layer (virtual layer)

The cognition layer is the brain of the architecture, with sophisticated simulation and emulation tools. It allows real-time monitoring, sustainability assessments, and predictive decision-making using historical data and live updates from the physical twin. Tools such as Siemens Tecnomatix Plant Simulation and AI algorithms enable users to perform virtual testing, optimize, and analyze scenarios. This layer integrates with immersive technologies such as AR and VR, providing interactive visualizations and intuitive control of DT. For instance, VR simulations can restore the entire manufacturing floor, where engineers can test configurations without actual physical changes (51).

Siemens Tecnomatix contains many tools that find usage in all the different layers within the six-layer architecture. Its emulation and simulation layers benefit mainly from (21). Plant Simulation is a crucial tool that enables logistic system modeling, simulation, and optimization by providing a digital, holistic replica of manufacturing processes. It focuses on material flow simulation to create real-world scenarios for performance evaluation. Simcenter, a product from Siemens, offers physics-based modeling and simulation tools that help optimize designs and processes to achieve an efficient DT. Additionally, Teamcenter, a product lifecycle management software, facilitates the connection and synchronization of data within a DT, enhancing interactivity within the six-layer architecture. Integrating these tools within the six-layer architecture allows organizations to monitor, analyze, and optimize their processes efficiently, thus significantly improving productivity and effectiveness across various industries (52).

The 5C Model is about strategic marketing since it analyzes the company, Customers, Competitors, Collaborators, and Climate to help businesses position themselves. However, the Six-Layer Architecture organizes generative AI systems development into six layers that guide implementing technology. While the 5C Model assesses internal strengths and external market conditions, the Six-Layer Architecture addresses technical capabilities in AI. The former is accessible for small to medium enterprises, while the latter requires specialized knowledge, making it suitable for advanced technology organizations (53).

### 2.5 Working of DT

DTs have proven valuable tools in multiple industries, revealing their potential to improve operations, enhance decision-making, and provide personalized medical care. This literature review reports five different models of working on digital twins designed in other years, showing these tools' evolution, application, and influence. The Apollo 13 DT (1970) case study is widely regarded as one of the first practical DTs (54). In the mission, simulators were used to represent the spacecraft systems, allowing mission control to test various scenarios and develop solutions to critical problems; among them was an oxygen tank explosion. This model allowed for real-time data analysis and simulation of decisions necessary for the mission's successful navigation back to Earth (55).

In 2017, another working illustration of Mayo Clinic's Patient-Specific Digital Twins (DTs) was created by integrating several data sources to construct models aimed at enhancing diagnosis and treatment for patients. This model synthesized data from medical imaging, genetics, and wearable technology to simulate individual physiologies, allowing healthcare providers to better tailor treatments for each patient. The impact on personalized care has been significant, with improved outcomes particularly noted in cardiac surgery planning (22). The Siemens Virtual Power Plant In 2018, Siemens implemented a DT in the form of a virtual power plant, which integrated various separated energy sources, like wind and solar. This model simulated the entire power generation system, optimizing renewable energy resources' management and operational efficiencies (56). The DT allowed for real-time performance analysis, enhancing the stability and sustainability of energy production (57).

In its 2021 DT Factories, BMW enhanced production efficiency by utilizing digital twins (DT) in its manufacturing processes. The company developed virtual replicas of its manufacturing facilities, simulating production flows and managing logistics in real time (58). This model enables BMW to predict disruptions and optimize manufacturing processes, thus reducing planning time by nearly a third. The initiative aligns with BMW's sustainability and digital transformation goals.

The most recent, 2023, DT for Drug Discovery was held at the University of Cambridge, where the recent initiatives at the University of Cambridge focused on creating DTs to assist with drug discovery (59). This model uses computer simulations and digital versions of cell processes to replace traditional lab experiments with virtual ones. It helps in assessing the impacts of drug interactions and predicting therapeutic outcomes before clinical application, significantly decreasing costs and time associated with drug development (60).

The development of DT parallels the increasing application of these models in multiple industries, such as healthcare and manufacturing. Each model is designed with features unique to its specific application, enhancing its predictability, efficiency, and personalization. As this technology develops further, the strategic benefits are likely to increase with its use across different disciplines.

DTs are the advanced digital representations of physical objects, systems, or processes, allowing for real-time simulation, analysis, and monitoring. They integrate information gathered from various sources about the performance and behavior of their physical equivalents throughout their lifetime (61). DTs are transformational tools that combine data collection, advanced modeling, real-time monitoring, predictive analytics, and continuous improvement to optimize systems and processes. They compile information from sensors, IoT gadgets, and previous sources to provide a digital replica of physical entities through 3D modeling and physics-based simulations (62). The digital models enable real-time monitoring, anomaly detection, and performance optimization with AI-driven analytics. Predictive analytics further strengthens decision-making by simulating scenarios and predicting maintenance needs, reducing downtime and costs. The DT is iterative in nature and ensures continuous refinement with new data, delivering more accurate insights and operational efficiencies. DT is a transformative technology that bridges the physical and digital worlds. Integrating data continually, creating dynamic simulations, and offering real-time insights allow organizations to promote innovation, increase operational effectiveness, and improve decision-making (63, 64).

### 2.6 Application of DT

DT technology is transforming various industries by making it possible to create digital copies of actual entities. DT technology and AI are changing healthcare by enabling precision medicine, advanced diagnostics, and personalized treatment planning (65, 66).

DTs are revolutionizing drug discovery, precision surgery, and disease modeling in healthcare. Through simulations of biological processes and patient-specific responses, drugs can be sped up in the manufacturing process, saving time and reducing the expenses for clinical trials. Virtual trials can test how effective and safe drugs are by trying them across various populations with no physical trial. Precision surgery is also realized through DTs, as the fine anatomical details allow surgeons to prepare for even the most delicate procedures and give them real-time input during surgical operations. DTs in disease modeling replicate diseases like diabetes and cancer, making treatment plans customized and predicting individual responses to therapies. For instance, Siemens Healthineers applies digital twins to model the reaction at the organ level, which helps optimize treatments for critical illnesses such as cancer. These advancements are improving patient results while cutting expenses and enhancing personalization in healthcare.

Digital twins (DT) are transforming manufacturing operations by providing real-time insights, enabling predictive maintenance, and facilitating process optimization (67–69).

Manufacturers monitor the production line parameters like temperature, pressure, and speed for efficiency in operations. They can detect anomalies that would signify defects through digital twins. DTs also offer predictive maintenance that identifies equipment failures before their occurrence to avoid downtime and maximize reliability (70). Consider Rolls-Royce's “IntelligentEngine” initiative. This approach improves the reliability of products and enhances customer experience by ensuring optimal performance.

DTs revolutionize project management in construction by simulating today's situation. These virtual models enable companies to test design changes, predict the impact of weather disruptions, and plan for emergencies without disrupting the construction process. DTs also optimize the use of resources, minimize waste, and help in more sustainable building practices. For example, London's Crossrail Project made use of DTs to envision outcomes, monitor progress, and address potential issues, thus smoothing workflows and resource allocation (71). Apart from efficiency, DTs enable energy consumption monitoring and eco-friendly design practices (72).

DTs are being integrated into urban planning to pave the path to smart cities, where technology and connectivity improve infrastructure and quality of life. Traffic flow, energy use, and emergency response plans can all be simulated and optimized using DT. For instance, cities can decrease congestion through DT to optimize the timing of traffic signals and predict bottlenecks. Environmental monitoring helps cities better manage air quality, waste systems, and carbon emissions to support sustainability. An example is Singapore's Virtual Singapore Platform, a holistic digital replica of the city-state. It promotes urban planning, infrastructure development, and environmental sustainability by integrating diverse data sources and providing a blueprint for future cities.

Amsterdam's first-ever 3D-printed pedestrian bridge, a 12-meter-long steel structure, has a DT created through sensors that track this structure's performance under stress. Led by the Turing Institute, this project provides crucial data on the safety and strength of 3D-printed structures, which will enable future engineering advancements. Tata Steel is also leveraging DT technology to revolutionize steelmaking by enhancing efficiency, sustainability, and innovation. Key initiatives include stabilizing the novel HIsarna process, which promises lower emissions and energy use, as part of its commitment to the EU's 2050 emission reduction targets. Real-time digital replicas enable the company to identify and resolve process inefficiencies cost-effectively, with plans to scale HIsarna in India. This approach exemplifies how AI, machine learning, and simulation-based tools can transform traditional industries and accelerate sustainable advancements (73).

DTs are revolutionizing various industries by enabling enhanced efficiency, innovation, and decision-making. In healthcare, DT facilitates personalized medicine by integrating patient data to tailor treatments, improve surgical training through virtual simulations, enhance diagnostics with advanced imaging analysis, and accelerate drug discovery by modeling drug effects on virtual patients (62–64). In manufacturing, they optimize operations with predictive maintenance, preventing costly downtime, and enable efficient product development by simulating scenarios to address issues early in the design process (65).

The aerospace and automotive sectors benefit significantly, with DTs used for predictive analytics to enhance flight safety and engineering reliability in aerospace and to refine autonomous vehicle development by simulating actual situations in the automobile sector (66).

**Figure 5:**
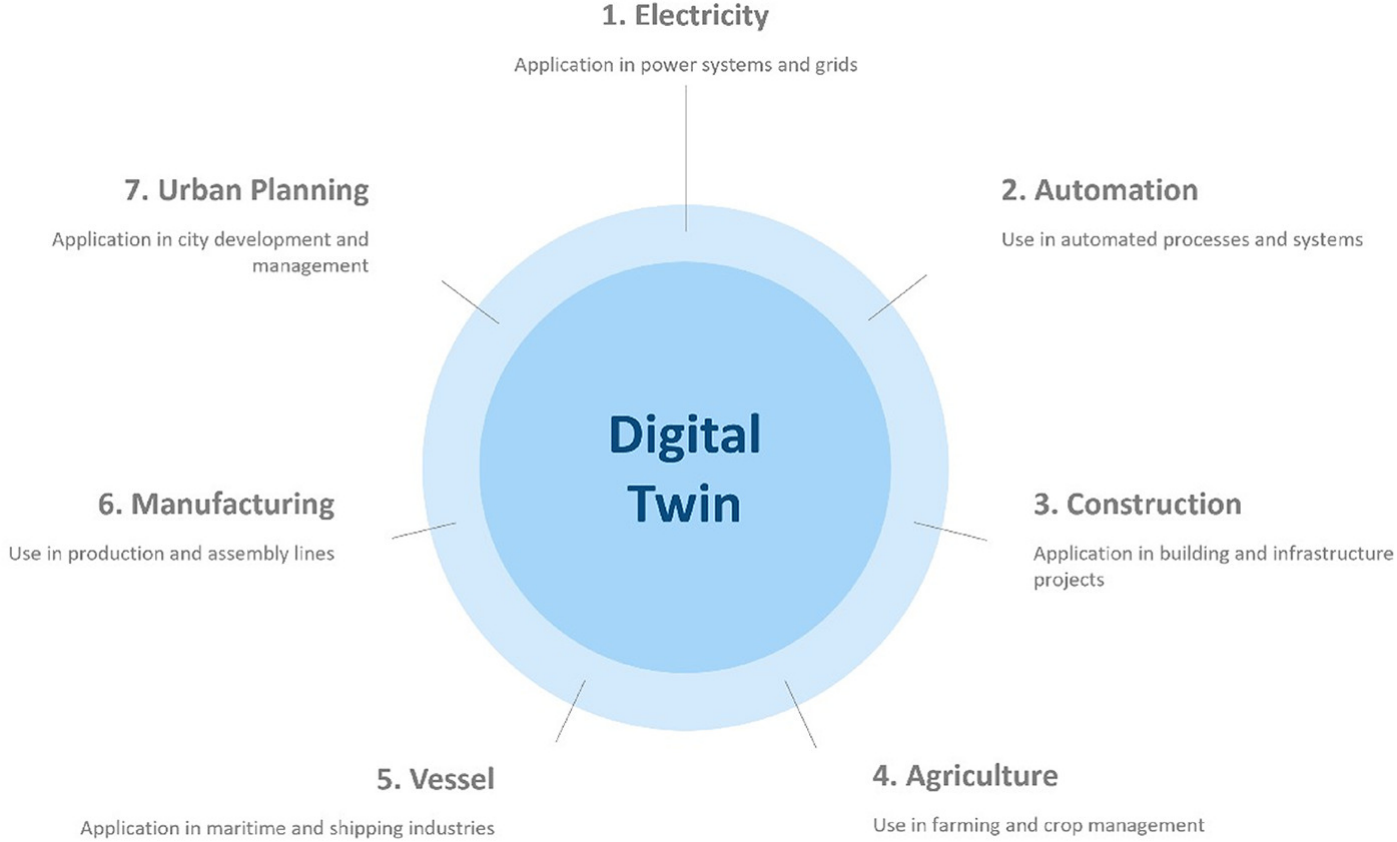
Dt applications for enhanced efficiency.

In smart cities, DTs combine IoT and geospatial data for urban planning, optimizing resource allocation, and infrastructure development. They also aid government agencies in emergency responses by simulating disaster scenarios and monitoring environmental factors like air quality, fostering sustainability, and improving living conditions (67, 68). By bridging the physical and digital worlds, DT drives innovation and solves complex challenges across domains (74, 75). Figure 5 depicts the DT applications for enhanced efficiency.

## 3 Personalized medicine (PM)

PM is also called precision medicine, a new medical strategy that adjusts medical treatments and therapy to the unique needs of each patient (76). The underpinning theory of this method is that treatment should not be “one-size-fits-all” but tailored explicitly according to genetics, environment, lifestyle, and unique individual clinical data (77). Personalized medicine, or PM, refers to medical intervention tailored to every patient's needs. Precision medicine is that treatment that uses personal data, like genetic characteristics and environmental and lifestyle factors, to enhance the identification, diagnosis, and management of disease (78). While precision medicine holds significant potential, data-sharing practices still face challenges regarding patient privacy and equitable access to care (79). It is a strategy that prevents, detects, and treats illnesses based on understanding an individual's genetics, proteins, and environment (80).

To evaluate individual risks and customize PM, or personalized genomics, precision medicine employs patient-specific profiles that combine genetic and genomic data to prevent and manage disease (81). Clinical and environmental factors play a significant role. This approach allows for a more individualized health experience, considering the differences in genetics, environment, and lifestyle that can impact health outcomes. By amalgamating these aspects, PM aspires to enhance preventive, diagnostic, and therapeutic practices (82).

### 3.1 Contribution of personalized medicine in disease prevention, diagnosis, and treatment

Tailored medicine can enhance illness prevention, diagnosis, and treatment in several ways. For instance, medical professionals can determine a patient's vulnerability to particular illnesses by looking at their family medical history (83). In some cases, a patient's family medical history may require genetic testing to determine if they could benefit from specific preventive care. Finding the mutation causing Lynch syndrome for those who have a family history of the illness, genetic testing might assist direct screening choices. For those who test positive for the mutation, routine and methodical testing for indications of precancerous growths in the colon can aid in the early diagnosis of sickness and possibly save lives (84). PM is often synonymous with targeted therapy, a treatment method focused on drugs that aim at specific molecules essential for the growth and proliferation of harmful diseases. Because PM is rooted in each patient's distinct genetic composition, it is progressively addressing the shortcomings of conventional medicine. Here is a table highlighting key events in the development of personalized medicine from its initial stages to the present (78).

### 3.2 The need for PM

Even though DNA remains consistent across different cells, the gene activity in organs functions (85). New technology like gene-expression microarrays allows us to study many genes at once, helping us tell apart gene activity linked to cancer from normal gene activity. Conventional medical practices have relied on cohort-based epidemiological research for many years, which typically ignores individual genetic differences and draws most results from a population perspective. When creating a treatment plan, modern customized medicine considers the patient's genetic profile and medical history. On the other hand, social background, lifestyle decisions, and the patient's family history determine traditional personalized treatment (86).

Current PM focuses on targeted therapies. For effective targeted therapy, it is crucial to know the altered biological pathways and components contributing to cancer (83). Advantages with PM include improved disease prevention and early detection, cost-effectiveness, minimization of adverse drug reactions, and tailored management plans (87). Historically, the traditional method of personalization has been based on a “reactive” approach: a physician assesses the patient, makes a diagnosis based on the observed symptoms, and prescribes medication. Unlike earlier treatments, modern treatments involve analyzing the patient's genetic history and family background for proper treatment (78). Genetics is the most significant component of PM since it gives healthcare professionals treatments and interventions based on a patient's genetic composition. This tailored approach enhances healthcare strategies regarding careful drug choice and dose adaptation. It may eventually improve therapy but reduce undesirable side effects (88). Table 2 outlines the origins and evolution of PM.

**Table 2:** Origins and evolution of PM.

Sr. no	Year	Event happened	Findings related to PM	Reference
1.	1869	Discovery of DNA by Friedrich Miescher	The identification of DNA laid the groundwork for future genetic research, essential for understanding genetic variations critical in PM.	(89)
2.	1896	Smallpox vaccination: Edward Jenner develops the smallpox vaccine, marking the beginning of personalized medicine.	First vaccine developed, paving the way for PM	(90)
2.	1901	Identification of the ABO blood group system	This discovery led to understanding individual biological differences, a foundational concept in PM for safer blood transfusions and disease susceptibility.	(89)
3.	1902	First account of recessive inheritance by Sir Archibald Garrod	Garrod's work linked genetic inheritance to disease susceptibility, establishing early principles that would later be vital in understanding genetic disorders in PM.	(89)
4.	1950	Emergence of pharmacogenetics	Researchers began examining how individual genetic differences affect drug responses, promoting the idea of tailoring medications to individual genetic profiles.	(91)
5.	1990	Launch of the Human Genome Project	A key component of PM techniques is the identification of specific genes linked to diseases, which was made possible by the massive genetic data provided by mapping the human genome.	(92)
6.	2003	Completion of the Human Genome Project	The completion resulted in a complete human genome reference sequence, facilitating new diagnostic tests and targeted therapies based on individual genetic profiles.	(91)
7.	2010	PM gains momentum: Customised medicine gains widespread recognition, with increased investment in research and development.	Increased recognition of PM, enabling further research and development	(93)
8.	2015	Food and Drug Administration (FDA) approval for genetic tests guiding drug prescription	Genetic testing began to be integrated into clinical practice, allowing personalized dosage adjustments for certain drugs, enhancing treatments’ efficacy and safety.	(94)
9.	2018	Launch of the NIH's All of Us Research Program	This initiative aimed at gathering diverse health data to understand health disparities better and enhance personalized medicine by identifying patterns associated with various diseases among populations.	(95)
10.	2020	PM becomes mainstream: PM becomes increasingly integrated into clinical practice, with widespread adoption of genetic testing and targeted therapies.	Widespread adoption of genetic testing and targeted therapies, enabling PM	(96)
11	2021	Increased FDA approvals for personalized therapies	More therapies targeting genetic variations have been approved, expanding patient treatment options based on genomic testing and individual characteristics.	(97)
12.	2024	Ongoing integration of AI in PM	Using artificial intelligence to analyse large datasets improves patient-specific treatments’ accuracy, enhancing predictive modelling for health outcomes.	(98)

### 3.3 Benefits of precision medicine

By ending the shortcomings of traditional treatment, personalized medicine customized to an individual patient's unique genetic profile is revolutionizing healthcare. It facilitates the shift from reactive to proactive treatments, enabling medical practitioners to improve early diagnosis, predict disease susceptibility, and act before the disease worsens. PM improves treatment outcomes by tailoring disease-prevention strategies and recommending more potent drugs while avoiding those with known side effects. Eliminating trial-and-error inefficiencies also reduces the length, costs, and failure rates of pharmaceutical clinical trials, lowering healthcare expenditures and improving patient care.

### 3.4 Current approaches to PM

PM enhances the accuracy of health care provision by utilizing an individual genetic profile. It has significantly increased the probability of correct diagnosis, effective treatment, and adverse drug effects. With the identification of appropriate strategies for treatment, PM is becoming a better prevention and control for many diseases, including tricky areas like oncology and rare genetic disorders (98).

#### 3.4.1 Pharmacogenomics

Pharmacogenomics studies how a patient's genetic composition can influence their reaction. To medication. It brings together pharmacology, which studies drugs, and genomics in understanding how genetic differences might impact drug metabolism, efficacy, and safety. PM helps provide treatment according to an individual's genetic makeup (99). In other words, the program enables targeted therapy for patients, which can significantly reduce adverse drug reactions and make treatments safer and more effective. It promotes the discovery of biomarkers for targeted therapy; it integrates genomic and lifestyle information to gain profound insights into health and accelerates clinical implementation by overcoming challenges in pharmacogenomics (100).

#### 3.4.2 Genetic testing and genomics

Examining a person's DNA is genetic testing for changes or mutations that can cause disease or impact health outcomes. The testing may uncover inherited conditions, predisposition to specific diseases, and information relevant to treatment decisions. Testing can identify variants that strongly influence how an individual responds to specific medications or the likelihood of a particular health problem (101). Personalized medicine uses genetics and genomics to target treatments, maximize drug effect, and predict risk for disease. This allows for enhanced therapeutic results and fewer adverse effects. Predictive risk assessments help in earlier interventions, and they help advance research by finding new therapeutic targets. The integration PM is made possible by the combination of lifestyle, environmental, and genetic factors. All these factors combine to revolutionize healthcare practice, making it more precise and tailored for patient care (102).

#### 3.4.3 Multiomics approaches

The multimomics approach combines and analyzes distinct biological data layers on genomics, transcriptomics, proteomics, and metabolomics to better explain intricate biological systems and disease mechanisms (88). Multiomics techniques help personalize medicine by bringing together data from genomics, transcriptomics, proteomics, and metabolomics to create tailored treatment plans, improve understanding of diseases, and better categorize patients. Such a holistic approach creates predictive models that cover the trajectory of disease progression and advances drug development by identifying biomarkers through targeted therapies (103). With the integration of multiomics data, PM allows for far more accurate diagnostics, treatments, and prevention procedures, improving patient health and creating a future healthcare system that is at its best (104).

#### 3.4.4 Data science and AI

Data science is a discipline that combines principles from statistics, mathematics, and programming with knowledge from the problem domain to glean insights and know-how from both structured and unstructured data (105). AI refers to computing systems that use human intelligence in simulating work to enable computers to perform functions based on human intelligence processes (105). PM, now driven by data science and AI, connects massive datasets of information to boost healthcare delivery in predictive analytics and genomic analysis coupled with better diagnosis accuracy. AI-driven tools enable tailored treatment plans, patient stratification, and early interventions, improving outcomes and reducing side effects. Continuous learning systems refine treatments based on real-world evidence, while resource efficiency is optimized through targeted interventions. These advancements promise a future of more individualized, effective, and resource-efficient healthcare, significantly enhancing patient care and outcomes (106).

As PM progresses, it underscores the necessity of tailoring healthcare approaches to individual characteristics, clearing the path for more creative and efficient patient care approaches in modern healthcare systems (88).

### 3.5 Limitations of traditional PM methods

Traditional PM faces several challenges, including limited genetic diversity in research, high costs, and ethical concerns related to data privacy. The complexity of implementation, requiring advanced technology and specialized expertise, further complicates its clinical integration. Accessibility is hindered by the high cost of genetic testing and personalized treatments, while the psychological impact of genetic risk predictions adds another layer of difficulty (107). Additionally, proving the efficacy of customized therapies over traditional methods is a slow process, and managing the vast amounts of data remains a significant hurdle. These limitations emphasize the need for continued innovation to overcome these barriers and enhance personalized healthcare (88). The economic relevance of PM presents significant challenges.

## 4 DTs in PM

**Figure 6:**
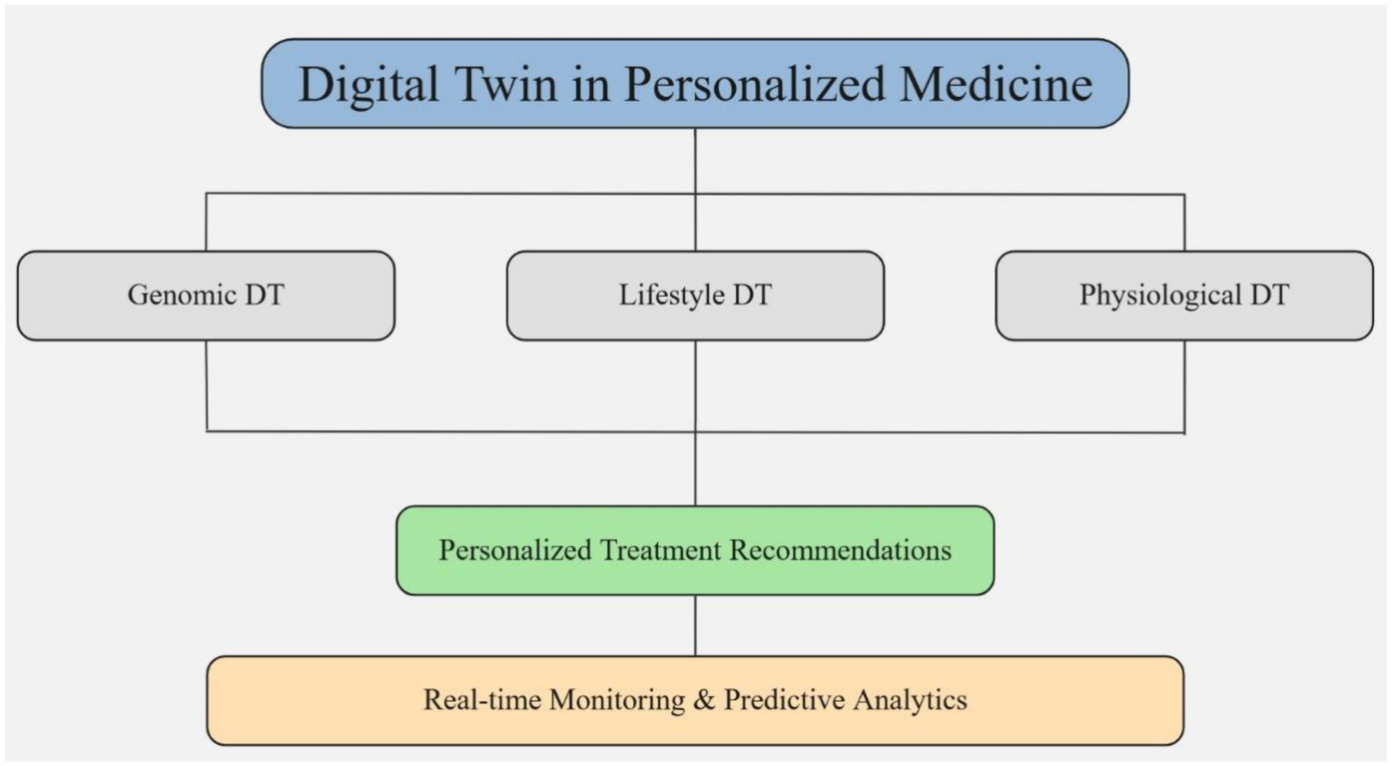
Dt in PM.

Incorporating DT into PM improves patient outcomes and advances healthcare innovation by fostering a more proactive and accurate approach to health management and boosting individual care (76, 108). DTs inherently combine multiple components, including a physical entity (the patient), a virtual model, and a bidirectional connection that allows real-time data exchange. This architecture supports dynamic simulations, allowing clinicians to visualize and predict patient responses to various interventions. Through continuous monitoring and integration of various data streams, such as EHR, wearable devices, and lifestyle factors, healthcare professionals gain comprehensive insights into a patient's health status, enhancing clinical decision-making (109). Figure 6 illustrates the application of DT technology in the context of PM.

### 4.1 Synergistic integration of digital twin technology with personalized medicine

The combination of DT technology and personalized medicine represents a revolutionary change in the conception and provision of healthcare. DTs offer the technological framework to continually model and improve medical treatments in real time, whereas PM concentrates on customizing medical care based on a patient's genetic profile, surroundings, and lifestyle. When combined, they create a dynamic feedback system that enables physicians to make patient-specific, data-driven decisions that change as new information becomes available (9).

The key part of this integration is that DTs can bring together different types of data into a single, virtual model of a patient, which includes genetic information, medical images, data from wearable devices, electronic health records, and behavioral information. Based on each patient's distinct physiological and molecular profile, this computerized model can predict treatment outcomes, simulate the evolution of the disease, and determine the best therapeutic approaches (17).

For instance, oncologists may create a digital twin to mimic the effectiveness of different chemotherapy regimens prior to administration, utilizing tumor imaging data, genetic mutations, and prior treatment responses (17, 19). Cardiologists can predict issues or make dynamic medication adjustments by using wearable technology to send real-time physiological signals (heart rate, blood pressure, and ECG) into a cardiac DT.

Additionally, DT technology enhances the potential of other cutting-edge personalized medicine tools. ML continuously refines these models based on fresh patient data, while AI algorithms can process large datasets from the digital twin to create prediction models for illness outcomes. In a similar way, DTs can improve simulations with very detailed accuracy by using various types of biological data, like proteomics, metabolomics, and genomics, by working together with bioinformatics tools.

This collaboration extends beyond diagnosis and therapy. By simulating patient cohorts in clinical trials, DTs can shorten recruitment times, customize trial designs, and find potential responders to experimental treatments. With digital therapies, DTs can modify behavioral interventions in response to ongoing monitoring, providing patients with chronic illnesses like depression or diabetes with personalized feedback.

Despite these developments, challenges still remain. Technical obstacles that need to be overcome include computing demands, real-time data integration, and interoperability among health systems. Equally important are ethical issues with algorithmic bias, consent, and data privacy. However, a new era of predictive, preventive, and fully personalized healthcare is anticipated to be fueled by the synergistic combination of DTs and PM as AI, IoT, and cloud technologies advance (25).

### 4.2 Conceptual framework for integrating DT in PM

The framework that conceptualizes the integration of DT technology in PM includes several essential components that collaborate to improve patient care and results. The work of Hossein Hassani et al. (110) The physical entity refers to the patient or individual within a healthcare context. The five aspects of the DT model in healthcare, as well as the virtual twin, the digital equivalent of the patient or human, are all essential elements. DT data (a combination of patient information and analytical insights from a digital model), services (the capabilities provided by the DT, such as monitoring, modeling, simulation, validation, and optimization), and data connection (the channels for data transfer between users and the digital counterpart, integrating digital twin data and services) (111).

Another study, “A Systematic Review: DT in Healthcare” (112) and “DT Technology in Healthcare: A Basic Review” (113), describes a six-step process for using DT in healthcare, which focuses on enhancing personalized patient care by integrating real-time data. Data collection, the first phase, involves gathering information from wearable sensors that monitor various health metrics. Subsequently, data cleaning is done to confirm the correctness and integrity of data before entering into the data processing stage, where it prepares the data for analysis. The next step in the process is developing the ML model. The fifth step involves the development of the DT, which generates a virtual patient model that emulates their health status and scenario. The final step in the process, prediction and visualization, assists healthcare practitioners with educated decisions by visualizing the information obtained from a DT. For easy access and smooth integration with real-time data, historical medical records are stored on the cloud. This all-encompassing framework improves patient monitoring and custom therapy and promotes proactive healthcare management, ultimately optimizing patient outcomes and boosting the overall efficiency of health care (114).

This framework model of DT in PM is complex and multifaceted. Tianze Sun et al. (115) have proposed a virtual representation of every patient to enable accurate diagnosis and well-structured treatment strategies. Five main components are integral to this framework: the physical entity, virtual model, connections, DT data, and service. The combination of DT technology with medical equipment and auxiliary medical devices creates an innovative platform and a novel approach to personal health management and healthcare services (116). This configuration facilitates the development of a virtual patient, formed from diverse data sources including medical imaging (such as CT, MRI, and ultrasound) and wearable devices. Biochemical markers and other components are also included. DT technology and high-resolution patient models can be used for simulations and big data processing. This technology can pinpoint precise treatment targets, identify appropriate medications, and determine optimal therapies, thereby achieving precision medicine, personalized treatment, and intelligent healthcare (117). The writer Kang Zhang (118) explores the advancement of a DT platform in healthcare, which consists of a four-phase roadmap that boosts functionality and complexity. The initial phase is static twins, which focuses on digitizing tangible objects, while the next phase, progressive twins, integrates real-time data for dynamic modeling. Operational twins allow continuous interaction between the virtual and physical environments, enabling real-time monitoring and adjustments (such as in insulin delivery). Lastly, autonomous twins represent a fully connected system where virtual models operate on their own but still interact with the real world, which could change areas like precision medicine, surgeries, and drug development.

**Figure 7:**
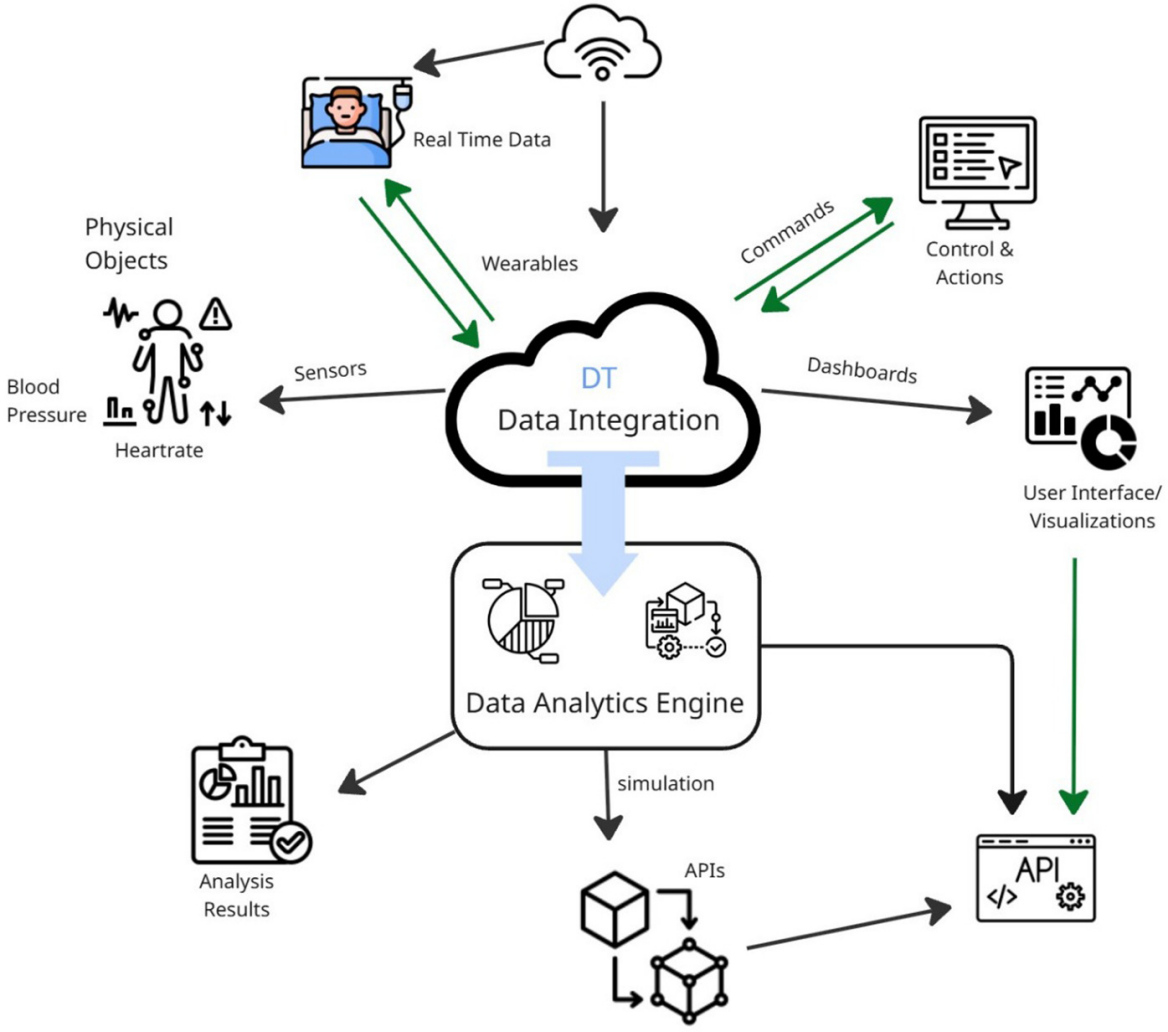
Architectural framework of the DT system.

The concept of a Digital Human Twin (DHT) is introduced as a detailed virtual representation of a person, incorporating anatomical, physiological, and preferably cognitive aspects. It facilitates two-way data interchange between the physical person and their DT, enabling tailored actions based on predictive models and real-time updates (119). To create a DHT, advanced data analysis and tools are needed to handle complicated biological information from different areas, such as multi-omics sciences, body structure details, and dual-purpose data. Access to extensive Excellent collections of medical data are essential for the accurate development of DHTs, employing AI and DL techniques to improve simulations. The connection between different healthcare systems requires standardized data representations and interoperability protocols to ensure a seamless information-sharing process (120). The DHTs can be realized with collaborative efforts by healthcare professionals and researchers and by addressing various ethical issues regarding data privacy, consent management, and more. These are essential to building confidence and protecting confidential health information (121). Figure 7 shows the architectural framework of the DT system.

### 4.3 Real-time monitoring and feedback mechanisms

The integration of real-time tracking and feedback systems in digital twins significantly enhances the personalization of medicine. Using continuous data updates and predictive analytics, healthcare professionals can provide interventions that improve patient outcomes and optimize care delivery (76, 122). The impact of DT in revolutionizingng customized healthcare is becoming increasingly exciting as technology advancesDoctors can apply DThieve accurate and timely diagnosis. DTs may help in differential diagnosis and reveal patterns that traditional diagnostic methods may miss by simulating different diagnostic scenarios through the analysis of patient data and symptoms. This enhances diagnostic accuracy, reduces errors, and makes it easier to intervene early, leading to more individualized and effective treatments (123).

The coThe combination of DT with real-time data from IoT devices, wearable technology, and remote monitoring tools enables continuous patient observation.king physiological parameters, vital signs, and more health-related information, DT can spot early signals of decline or abnormalities. This ability enables medical professionals to avoid problems, take preventative measures, and alter treatment regimens. Real-time monitoring with DT is particularly beneficial for individuals with chronic illnesses since it minimizes the need for hospital visits and enables remote patient care (124).

DTs enable patients to be more proactive in their health managtheireir treatment plans, progress tracking, and tailored health insights will make this kind of involvement easier (125). The outcome will be better adherence to treatment regimens, lifestyle modifications, and self-management techniques (13). Additionally, through shared decision-making and patient-focused approaches to care, DT can support improved interaction and cooperation between healthcare providers and patients (110).

These systems can anticipate the onset of diseases and treatment outcomes by using machine learning and predictive analytics approaches (126). DTs can provide continuous care by safely sharing patient data across healthcare settings and providers. Such sharing promotes Such sharingdinated care, reduces pointless testing, and lowers medical errors by guaranteeing that all participating healthcare professionals can access the most up-to-date and thorough patient information (127). DTs can also help the healthcare team communicate and collaborate better, improving the care of patients in general (110).

### 4.4 Data collection and management

DT technology promises to improve patient care by utilizing real-time data integration, advanced analytics, and customized insights (128). Various types of data sources exist, including genomic, phenotypic, lifestyle, and environmental factors. These tools empower healthcare professionals to collect and analyze extensive patient information from multiple sources, such as EHRs, medical devices, wearables, and genetic data (123, 129). Through the use of advanced data processing methods and reliable storage solutions, DTs signify a significant progression in personalized medicine. Such technologies can considerably improve patient care by facilitating customized interventions and offering predictive insights. Nevertheless, it is essential to tackle ongoing issues related to interoperability, data quality, and ethical considerations to effectively integrate DTs in clinical environments (130). By combining and analyzing this information, DT comprehensively views each patient, enabling healthcare providers to formulate tailored treatment plans (131, 132).

## 5 Use cases DT use cases in healthcare

### 5.1 Enhancing surgical precision and safety with patient-specific 3D maps

Cydar is a company that specializes in surgically enhanced intelligence. Its EV Maps program transforms surgical procedures by using cutting-edge technology such as cloud GPU processing, computer vision, and ML. This advanced platform enables decision-making and enhances visualization during procedures and the course of therapy (133). Cydar uses these developments to assist medical practitioners in developing accurate, patient-specific 3D maps. All surgical intervention phases used these maps, from preoperative planning and in-procedure navigation to postoperative evaluation (134). This technique of a digital twin in healthcare has numerous benefits associated with it, including reduced exposure of patients and surgery teams to radiation, acceleration of surgery processes, and high confidence of the surgical teams in the aptness of their treatments (135).

### 5.2 AI-driven insights for precision in heart procedures

The FEops HEART guide is a new technology that brings together digital twin and AI potential to transform the way structural heart procedure planning is conducted. It gives physicians critical insights into device sizing and positioning by creating virtual replicas of patients’ hearts, thus reducing procedural risks and improving outcomes. The U.S. has recently approved its use for Left Atrial Appendage Occlusion (LAAo) workflows. It merges AI-driven anatomical analysis with predictive simulation, thereby transforming cardiac imaging into a digital twin that provides highly valuable data-driven insights (136). In addition to providing insight into the device sizing and positioning, it has proven to have a 25% decrease in procedural complications and 15% better long-term patient outcomes. Additionally, it makes use of Robotic Process Automation.

(RPA) for better efficiency by streamlining the administrative and operational tasks to build an advanced healthcare ecosystem. This technology has successfully predicted the device-patient interactions, optimized the treatment of the patients, and improved the outcome of the cardiac procedures (137).

### 5.3 Enhanced diagnostics and hospital efficiency

DTs in healthcare allow for precise diagnostics by simulating patient-specific scenarios, identifying potential issues, and optimizing treatments. For example, hospitals use DTRs to model the distribution of resources and patient flow. This strategy has resulted in a 15% increase in bed utilization efficiency and a 20%–30% decrease in patient waiting times.

One of the top providers of orthopedic solutions and prosthetics is Össur. They create and produce individualized prosthetics using DT technology. Össur leverages DT technology to create custom-made prosthetics. By simulating design iterations using a patient's DT, they achieve a 95% fit accuracy on the first attempt, enhancing patient comfort and reducing the need for costly adjustments (138).

### 5.4 PM and drug development

In medicine, DT enables tailored treatments by combining patient data such as medical history, genetic profiles, and health metrics. This approach has increased treatment efficacy by up to 50% while reducing adverse side effects by 30%–40%. Additionally, DTs accelerate drug development by simulating virtual patients, significantly shortening clinical trial durations.

**Figure 8:**
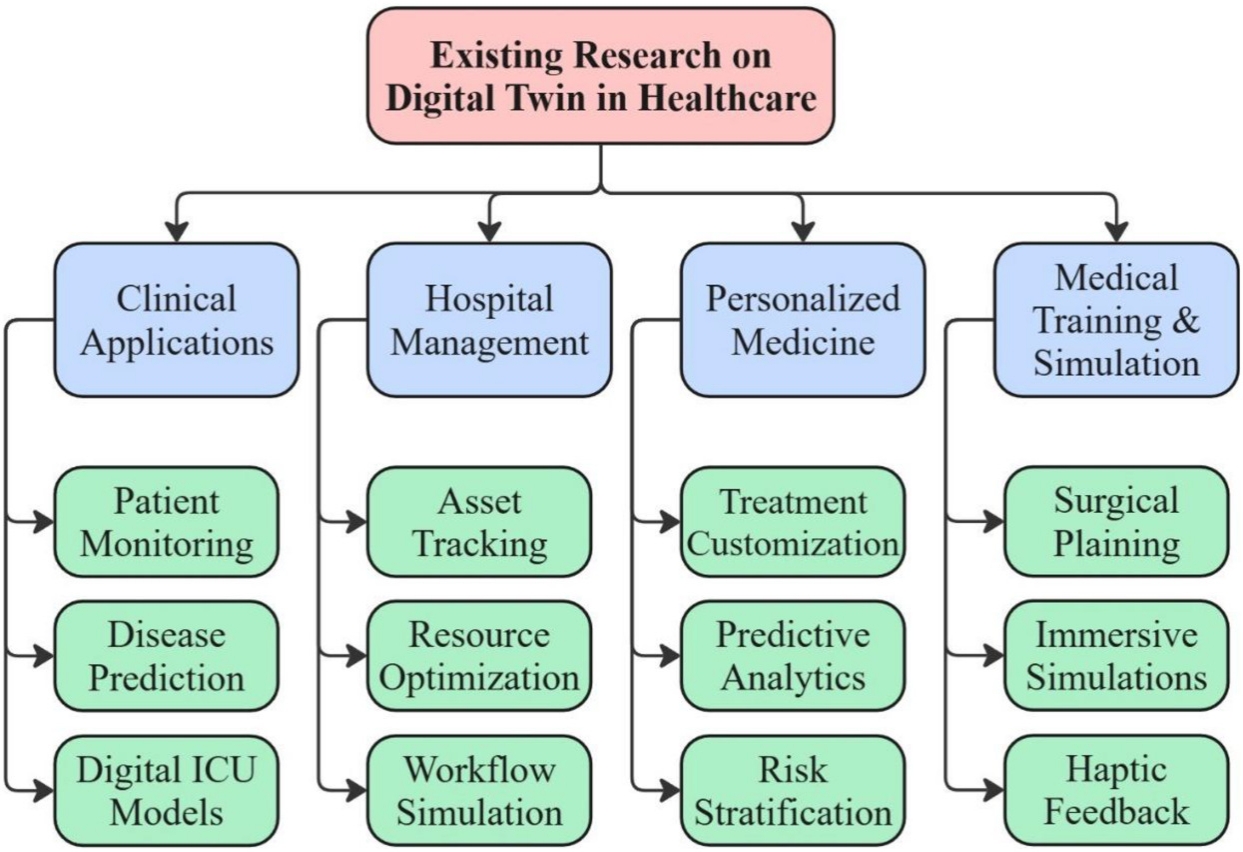
Major research domains of DT technology in healthcare.

Pfizer uses DTs to improve their efforts in cancer research and development. The goal is to learn more about the mechanisms behind cancer so that pharmaceutical companies can test new medications on cancer patients. Pfizer uses DTs to model cancer progression and test experimental drugs. This methodology has reduced preclinical testing times by 30%, allowing faster identification of promising treatments (139). It also ensures drug safety and efficacy before real-world trials, reducing overall development costs (140). Figure 8 summarizes the major research domains of DT technology in healthcare. It highlights four primary categories, such as clinical applications, hospital management, PM, and medical training and stimulation.

## 6 Benefits of DT in healthcare

**Figure 9:**
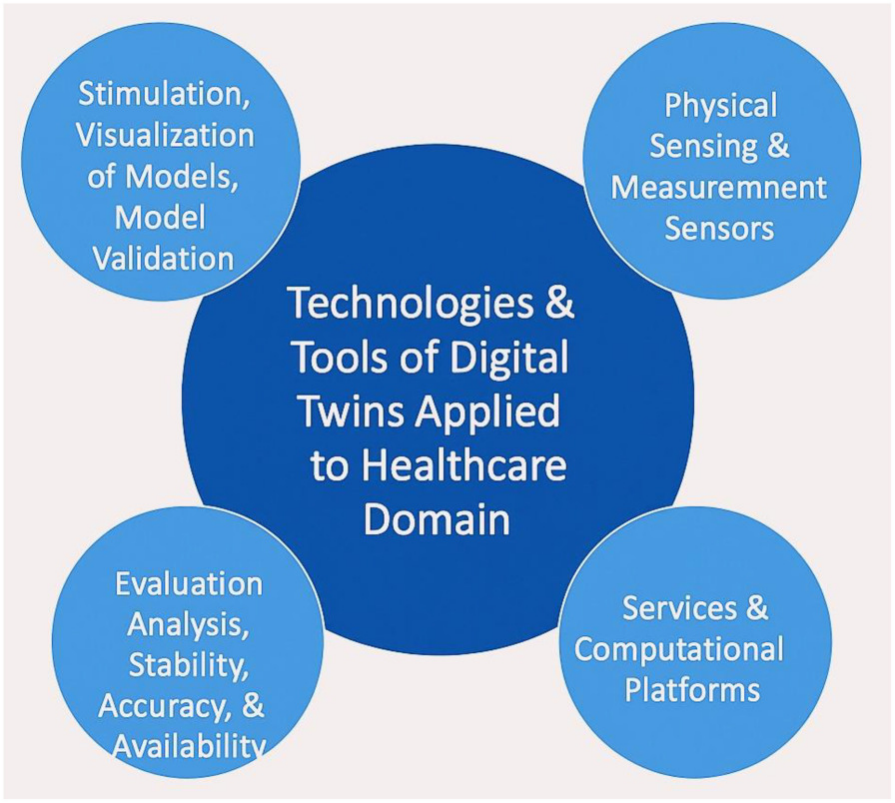
Presents the technologies and tools utilized in DT systems.

DT in healthcare is the most transformative approach that enhances patient care and operational efficiency. The significant advantages are that it allows PM, since the simulated response of an individual patient to different therapies can be displayed. Figure 9 presents the technologies and tools utilized in DT systems.

Consequently, it provides enhanced treatment plans (141). Predictive analytics and opportunities for healthcare issues and treatment outcomes through digital twins are possible, enabling proactive interventions and better management of chronic diseases (142). It also serves as a valuable resource for medical professionals, offering a risk-free environment to practice complex procedures on virtual patients (143). DTs improve operational efficiency by identifying inefficiencies in patient flow and resource allocation that eventually lead to decreased wait times (144). In drug development, they allow faster and safer testing of new therapies by simulating biological processes, thus accelerating the drug discovery (145). In addition, DT enables continuous remote monitoring of patients, allowing for timely intervention, particularly in the case of patients located in remote areas. Through optimizing processes and enhancing surgical planning by using detailed 3D anatomical models, DT contributes to better outcomes for patients with cost savings in healthcare delivery (146). Overall, integrating DT technology in healthcare significantly improves patient care and operational effectiveness (147).

Predictive analytics and preventive measures: DT enables the early identification of health risks and disease progression by combining a lot of data about a patient. This will then allow for timely treatments and preventive measures. By focusing on the potential issues and giving preventive measures, such an approach optimizes resource allocation to achieve improved patient outcomes.

Enhancement of Clinical Operations and Cost Efficiency: DTs provide useful information about patient numbers, demand trends, and resource usage, helping in effective resource allocation and enhancing operational performance. By supporting quality improvement activities and facilitating ongoing monitoring of clinical operations, they streamline clinical workflows and increase patient safety.

Training & Simulation: DTs can replicate actual settings to train healthcare personnel, especially in complex procedures and emergencies. Improved patient care and safety result from this training's skill development, decision-making ability, and professional collaboration.

### 6.1 DT of a healthcare facility

DT technology allows for establishing a virtual hospital and assessing its staffing, operational strategies, capacity, and care models to identify areas that need improvement, anticipate possible obstacles, and improve organizational tactics. Therefore, it is possible to use digital representations of hospitals to make facility replicas, which in turn allows:

By tailoring treatments to each genetic and health profile, DTs reduce adverse drug reactions by 30%–40%. Simulating virtual patients reduces preclinical and clinical trial durations by up to 30%, saving pharmaceutical companies millions in development costs. DTs, combined with AI, enhance diagnostic accuracy by 20%–25%, minimizing unnecessary tests and procedures.

Through better risk reduction and resource management, digital twins are revolutionizing hospital operations. DTs enable precise forecasts and flexible adjustments by leveraging historical and current data about hospital operations and outside factors like COVID-19 case counts or traffic incidents. This feature can help hospitals effectively monitor the usage of operating rooms, improve staff schedules, and detect bed shortages. All these changes decrease the costs of operations apart from increasing efficiency in the distribution of resources. For example, it has been established that with the use of DT in integrated process management, the amount of time a stroke patient should receive treatment is reduced significantly, proving to have benefits both for patient care and efficiency in the hospitals.

DTs are revolutionizing hospital operations by efficiently managing resources and reducing risks. The use of historical and real-time data concerning hospital functions and external influences, such as numbers of COVID-19 cases or traffic accidents, helps DTs provide accurate predictions and flexible changes. This capacity helps hospitals realize shortages in available beds, staff schedule optimization, and efficient operating room utilization. These improvements not only enhance efficiency in the distribution of resources but also reduce operational expenses. For instance, studies have shown that using digital twins in integrated process management greatly reduced the time to treat stroke patients, thus providing evidence of clear advantages in patient care and hospital performance. Besides resource management, DT plays a crucial role in risk assessment. By providing a virtual testing space, hospital administrators can analyze the effects of changes in critical areas such as staff availability, operating room timings, and maintenance schedules. This evidence-based approach guarantees that strategic choices are executed safely, minimizing disruptions and ensuring operational stability in a complex and vital healthcare environment.

### 6.2 DT of the human body

To develop tailored medicine and treatment regimens, DT also mimics organs, specific cells, or a person's genetic composition, physiological traits, and lifestyle choices. Collecting vital individual-level data, such as blood pressure and oxygen level, empowers individuals to monitor chronic conditions and provide essential information for more informed doctor-patient interactions. This personalized approach replaces generalized treatments derived from large samples with tailored simulations, allowing doctors to predict patient-specific reactions to various treatments. As a result, the accuracy and efficiency of treatment plans are significantly improved, leading to better patient outcomes and advancements in clinical research. In treatment planning, DT uses sophisticated modeling to represent the human body with high precision. This means doctors can diagnose pathologies even before symptoms manifest and test alternative treatment options, fine-tune preparations for surgery, and so on. DTs enhance decision-making, reduce risks, and improve procedural success rates by providing a virtual environment for testing scenarios. A pilot study on hospital digital twins improved operational efficiency by 15% and cut emergency department congestion by 20%. These innovations enable more precise, data-driven, and patient-centric healthcare solutions.

### 6.3 DTs for medicine and device development

Digital twins improve the design, development, evaluation, and tracking of innovative drugs and medical equipment in the healthcare industry. To crowdsource a virtual model of the heart, the Living Heart project was started in 2014. Through computer simulation, this team has created a validated DT model of the human heart that investigates drug interactions with the organ. A DHT is also being used to speed advancing the creation and enhancement of cardiac device architecture with the aid of new collaborations between Siemens Healthineers and Philips.

DT are essential in the advancement of both pharmaceuticals and medical devices. In drug development, DT for various drugs and chemical substances allows researchers to alter or redesign formulations by considering factors like particle size and composition, which enhances delivery effectiveness. Likewise, in medical device design, digital twins enable researchers to evaluate attributes of a device or analyze alterations in design or materials to understand how these changes would affect the outcome in a virtual world before real production. It reduces failure-related costs while enhancing the performance and final product's safety and quality.

Realistic anatomical models may be the contact point for medical students and surgeons, which improves procedural skills and minimizes errors. According to Medical Simulation in Healthcare, 2023, research demonstrates a 35 percent increase in procedural confidence when training using DT. Real-time monitoring of medical devices ensures timely maintenance, reducing equipment downtime by 25 percent and cutting repair costs by 30 percent. As technology advances, more DT models for organs are anticipated, which will improve patient outcomes, enable more precise and customized medical interventions, and streamline resource management in the healthcare industry (148).

## 7 Challenges of digital twins in healthcare

There are various obstacles to DT's use in healthcare. Integrating several sophisticated data sources such as electronic health records, medical imaging, and real-time monitoring devices, which typically have disparate standards and formats, is one of the most difficult tasks. Another significant concern is data security and privacy since digital twins will be dealing with sensitive patient information. Moreover, the computational power and advanced algorithms required for the accurate simulation of digital twins can be very resource-intensive (149). EHRs require data standardization and interoperability (150). EHR systems often employ various formats, coding standards, and terminologies, which complicates data integration. Interoperability problems happen because some systems are private or don't follow standards like Fast Healthcare Interoperability Resources (FHIR), and when data is inconsistent or missing, it makes DT models less reliable. There are also issues with the accuracy and validity of the models, which must be updated regularly with new data to remain valid (88).

### 7.1 Data security and privacy

The use of DT in healthcare requires large amounts of personal health information. This information is not only sensitive but also constitutes the most private information individuals own. Therefore, ensuring this information's security and privacy is paramount. Violation of data privacy can lead to issues such as identity theft or insurance fraud. In serious situations, a patient may face discrimination based on his health status. For example, if a patient's prognosis is unfavorable, an insurance provider may refuse to approve coverage for a new medication (50). Healthcare DTs rely on the successful accumulation of in-depth health data from individuals, including very personal health details. Privacy and security must be guaranteed at their best. Balancing the desire for access to critical health data without infringing privacy remains a major challenge. Data privacy and security are also a significant challenge for DT in healthcare, especially since most patient information is collected for analysis, including sensitive health data (33).

### 7.2 Data accuracy and completeness

For a DT to effectively present a patient's health requires a complete set of medical data. Thus, for effectiveness in the digital twins, the completeness and correctness of data are an absolute necessity (79). Data accuracy and completeness in healthcare depend on several key elements. A thorough medical history is essential for getting a patient's past conditions and treatments. Genetic information is crucial in identifying hereditary diseases, though ethical and practical challenges may limit availability. Lifestyle factors, including diet, exercise, stress levels, and substance use, also significantly impact health outcomes. Additionally, real-time health data from wearable technologies offer valuable insights, but their reliability is limited as patients may not always wear the devices consistently. Consequently, collecting and integrating such diverse data remains a significant challenge in healthcare.

### 7.3 Technical complexity and cost

Implementing digital twins in healthcare requires high technical expertise, continuous updates, and complex integration with existing IT systems, making it feasible mainly for advanced providers (151). The costs are substantial due to expensive technology, data handling, and the need for skilled personnel and frequent maintenance. Security measures and consulting experts further add to the financial burden. However, partnering with digital twin experts or outsourcing tech work can help reduce these costs, making implementation more manageable for healthcare providers (152). Economic barriers, such as high implementation costs, add another layer of complexity. Developing digital twins requires substantial investment in advanced technology, skilled professionals, and research, making it inaccessible for smaller healthcare providers. Concerns about return on investment can also deter stakeholders. Potential solutions include fostering collaborations to share costs across academic institutions, technological firms, and healthcare providers; offering government incentives such as grants and tax breaks; and leveraging cost-effective cloud-based platforms to reduce initial expenses (33, 153). Deploying DT solutions can be a significant financial burden, with costs for medium-scale implementations ranging from $100,000 to $500,000 (154).

### 7.4 Patient consent, engagement and scaling

Acquiring patient consent can often be difficult, primarily because they may not fully grasp what they are consenting to. The notion of DTs is intricate, leading to patients not fully comprehending its significance or benefits. Even if they do understand its functionality, they may raise privacy concerns. Furthermore, there are long-term implications associated with digital twins to consider. Collecting and updating patient data is necessary to ensure the DT accurately reflects the patient's health condition. Scaling the application of DT from a limited setting to a wider and more varied range of conditions can be daunting. These conditions can encompass various factors, including patient demographics and different health issues.

As technology advances, there is potential to permanently alleviate some of these issues. Additionally, increased competition among service providers will likely reduce costs (155).

### 7.5 Ethical considerations

There are several ethical issues with creating DT for the medical field. These difficulties include getting people's informed consent for the collecting and using of their data in the creation of DT, dealing with concerns about data ownership and management, protecting patient autonomy, and being aware of legal limitations. Maintaining healthcare fairness is also essential to prevent DTs from escalating health inequities. The development of health-related DT requires the establishment of ethical frameworks for informed consent, data anonymization, and responsible and secure data sharing permission to promote trust and preserve moral principles (156, 157).

## 8 Future prospects

The global DT technology market was valued at approximately $11.82 billion in 2023, having grown at a compound annual growth rate (CAGR) of 24.34% from 2018. The market is expected to grow from $11.82 billion in 2023 to $59.25 billion in 2028 at a CAGR of 38.04%. The market is projected to develop at a CAGR of 26.96% between 2028 and 2033, reaching $195.44 billion (158). Because it provides previously unheard-of insights into intricate systems and processes, digital twin technology is transforming industries worldwide (69). This innovative technology enables real-time monitoring, analysis, and optimization by producing virtual versions of real-world items, settings, or procedures (159). The future for digital twins in healthcare is highly exciting and intriguing. Technologies like 5G, edge computing, and blockchain are ready to play major roles. These technologies will significantly enable high-speed connectivity, facilitate the transfer of real-time data, and enhance the development of dynamic and responsive digital twin systems. Edge computing will take data closer to its source so that latency reduces and efficiency increases, which is especially pertinent for applications demanding instant analytics remote monitoring or surgical assistance. AI will be another powerful driver in DT evolution: Generative models such as generative pretrained transformers (ChatGPT) and the other advanced AI systems can optimize the predictive ability of digital twins by analyzing vast datasets, simulating patient-specific outcomes, and providing adaptive recommendations. These models can enhance personalization in diagnostics, treatment planning, and the development of drugs, making these digital twins more powerful and precise tools in healthcare delivery.

### 8.1 Emerging trends in DT technology

With its unparalleled insight and optimization potential, DT is revolutionizing several sectors. Its capacity to create virtual representations of tangible objects and procedures has significantly influenced performance, cost-effectiveness, and decision-making across several sectors, including manufacturing, healthcare, and smart cities (160).

This capability allows clinicians to model various treatment scenarios, forecasting how a particular patient will respond to a given therapy, which leads to more personalized and effective treatments. For instance, oncologists can use DT to model the behavior of a tumor and predict the effectiveness of different chemotherapy options for a specific patient based on that patient's unique cancer profile. Progress on data analytics, AI, and ML is continually upgrading the accuracy of these simulations; thus, identification of the best therapy strategies can be done for the wide population (161). Additionally, next-generation continuous streams of data based on the Internet of Things provide guidance for medical decisions. AI and ML algorithms process all this information to identify patterns and insights, making predictive analytics possible to predict patient decline and support timely interventions. Collectively, these technologies are enhancing the functionalities of digital twins and paving the way for more intelligent, responsive health care systems emphasizing patient-centric care and operational effectiveness, revolutionizing health care delivery (162).

### 8.2 Technological advancements

DT providers are beginning to weave emerging technologies such as AI and big data into their offerings to develop comprehensive DT capable of overseeing entire operations, like a whole factory. As more people start (75) using AI tools like ChatGPT, we expect that AI will greatly impact how digital twins develop, making them more efficient and increasing their market size. Blockchain represents another fascinating example of how emerging technologies can be combined with digital twins; its high levels of encryption can bolster the security of DT (163). Through cutting-edge technologies, DT in personalized medicine generates dynamic virtual representations of each patient, enabling customized medical therapies. Healthcare professionals can gather and analyze real-time data from clinical and environmental sensors by combining various data sources, such as IoT devices, artificial intelligence, and cloud computing. Such an approach allows them to provide insightful information that helps guide medical decisions (164).

In conclusion, rather than merely being a continuation of existing technological developments, the future of DT is an exploration of a world where sustainability, creativity, and responsibility coexist. Through revolutionary development, the field of DT technology has the potential to impact several sectors profoundly. By evaluating alternative strategies and modeling “what-if” scenarios, interactive DTs help businesses become more efficient and proactively handle challenges. Table 3 presents the technologies and tools utilized in DT systems.

**Table 3:** Presents the technologies and tools utilized in DT systems.

Sr. no	Technology with DT	Description
1.	AI-driven digital twinning	AI-driven DTs in healthcare use machine learning, NLP, and deep learning to model patient behaviours, enabling detailed simulations, accurate predictions, and personalized treatments by analysing structured and unstructured medical data.
2.	XR	Combining DTs with XR technologies like VR and AR creates immersive, interactive experiences, enhancing understanding, collaboration, and real-time guidance. This integration revolutionizes learning, problem-solving, and remote work, bridging physical and virtual worlds.
3.	Cloud-based solutions and DTaaS	Cloud-based technologies will continue to be utilized to give digital twins as a service (DTaaS) democratizes digital twin technology, making it accessible and affordable for businesses by eliminating costly infrastructure needs and providing secure data storage, processing, and flexible, remote access to robust digital twin solutions.
4.	Expansion into new domains and markets	DTs are expanding beyond early sectors like healthcare and aerospace to revolutionize transportation, retail, and energy industries by optimizing power systems, enhancing customer experiences, and driving innovation across complex challenges.
5.	Sustainability and Environment-friendliness	DTs will drive sustainability by optimizing resource use, reducing emissions, and aiding eco-friendly design in sectors like construction and smart cities, becoming crucial tools for a greener, more sustainable future.
6.	Combining Real-time Analytics with Edge Computing	Incorporating edge computing into DT will enable real-time analytics, swift responses, and low-latency decision-making, benefiting applications like industrial automation, smart cities, and autonomous vehicles while enhancing privacy and resource efficiency.
7.	5G Networking for Effective Data Transmission	The deployment of 5G will enhance DT capabilities, enabling real-time sync, remote control, and tracking, benefiting sectors like autonomous vehicles, manufacturing, and healthcare with faster, low-latency data exchange.
8.	Concerns about Data Confidentiality and Ethics in DT	As DT technology expands, addressing confidentiality, data ownership, and privacy becomes crucial. Businesses and legislators must ensure ethical use through regulations, encryption, and access control, fostering public trust and responsible adoption.
9.	Increasing Adoption of DTs	By 2023, 75% of enterprises embraced DTs to drive digital transformation, enhance remote work, improve customer experiences, and streamline development, while supporting sustainability, process mining, and risk analysis across diverse sectors.
10.	Evolutions in the Blockchain	By 2032, blockchain will be integral to DTs, enabling real-time analytics, enhanced security, and process optimization. Innovations like BlockTwins combine blockchain, process mining, and edge computing to advance automation, risk management, and compliance.

### 8.3 Long-term impacts

The evolutionary impacts of technologies, especially DT technology and personalized medicine, will significantly reshape industry standards and practices in the next decade. The integration of DT is expected to improve efficiency and provide better insights regarding system performance in all sectors, from manufacturing to healthcare. As the value of these virtual replicas continues to be acknowledged by organizations, they will implement new standards based on data-driven decision-making and real-time systems monitoring. It streamlines the processes and sets a new quality assurance benchmark, predictive maintenance, and sustainability practices in the industries, thus changing the basic operational protocols (15).

In precision medicine, the potential of genomics and AI will revolutionize patient care by driving the development of highly tailored treatment plans. Precision healthcare will require new rules for genetic testing and personalized treatment plans, making sure that treatments match each patient's unique genetic makeup and lifestyle. Additionally, as digital technology is used more in healthcare, it will help doctors monitor patients better and assess risks more accurately, allowing for immediate changes in treatment plans to improve results.

It would be interesting to see how things change in these industries in the next decade. Predictions say that this market for the DT would continue to expand, with the investments leading to enhanced capabilities and broader applications in sectors ranging from urban planning to environmental sustainability (165). At the same time, the market for personalized medicine is projected to increase enormously due to technological advancements and the demand for customized healthcare products, and there is an anticipation of a substantial increase in value by 2030. This will further augment the trend and facilitate greater incorporation and automation into operational processes. Technologies will enable industries to be innovative, connecting all stakeholders for collaboration, efficiency of data sharing, and optimal service delivery. In summary, these innovations will propel the industries towards better, more responsive, and more personalized practices, resulting in more technologically integrated and patient-centered approaches (166–168).

## 9 Conclusion

DT technology is emerging as a cornerstone of modern personalized medicine. By creating real-time, data-driven digital replicas of patients, DTs enable dynamic simulations of disease progression, treatment responses, and health outcomes. Their use enhances clinical decision-making, supports early diagnostics, and allows for individualized therapeutic strategies tailored to each patient's genetic, lifestyle, and physiological data. The integration of AI, IoT, big data, and machine learning within DT systems drives this shift from generalized care to precision medicine. However, the full-scale adoption of DTs faces notable challenges, including data privacy concerns, interoperability limitations, ethical issues regarding patient autonomy, and the complexity of integrating with existing healthcare infrastructures. Addressing these barriers requires robust regulatory frameworks, standardization protocols, and interdisciplinary collaboration. Looking forward, advancements in AI, cloud computing, and blockchain technologies hold promise for resolving current limitations. These innovations can enhance data security, processing capabilities, and system integration, paving the way for scalable, secure, and effective deployment of DTs in clinical practice. As research and investment continue to grow, DTs are poised to redefine healthcare delivery, enabling a future where predictive, preventive, and truly personalized medicine becomes the norm.
